# Liver Bile Acid Changes in Mouse Models of Alzheimer’s Disease

**DOI:** 10.3390/ijms22147451

**Published:** 2021-07-12

**Authors:** Harpreet Kaur, Drew Seeger, Svetlana Golovko, Mikhail Golovko, Colin Kelly Combs

**Affiliations:** Department of Biomedical Sciences, School of Medicine and Health Sciences, University of North Dakota, 1301 N Columbia Road, Grand Forks, ND 58202-9037, USA; harpreet.kaur@und.edu (H.K.); drew.seeger@und.edu (D.S.); svetlana.golovko@und.edu (S.G.); mikhail.golovko@und.edu (M.G.)

**Keywords:** bile acid, sex difference, Alzheimer’s disease, cholesterol, cytochrome P450

## Abstract

Alzheimer’s disease (AD) is a neurodegenerative disease characterized by progressive cognitive impairment. It is hypothesized to develop due to the dysfunction of two major proteins, amyloid-β (Aβ) and microtubule-associated protein, tau. Evidence supports the involvement of cholesterol changes in both the generation and deposition of Aβ. This study was performed to better understand the role of liver cholesterol and bile acid metabolism in the pathophysiology of AD. We used male and female wild-type control (C57BL/6J) mice to compare to two well-characterized amyloidosis models of AD, APP/PS1, and *App^NL-G-F^*. Both conjugated and unconjugated primary and secondary bile acids were quantified using UPLC-MS/MS from livers of control and AD mice. We also measured cholesterol and its metabolites and identified changes in levels of proteins associated with bile acid synthesis and signaling. We observed sex differences in liver cholesterol levels accompanied by differences in levels of synthesis intermediates and conjugated and unconjugated liver primary bile acids in both APP/PS1 and *App^NL-G-F^* mice when compared to controls. Our data revealed fundamental deficiencies in cholesterol metabolism and bile acid synthesis in the livers of two different AD mouse lines. These findings strengthen the involvement of liver metabolism in the pathophysiology of AD.

## 1. Introduction

Alzheimer’s disease (AD) is a progressive neurodegenerative disease characterized by progressive cognitive decline. The extracellular aggregation of amyloid β (Aβ) peptides and intracellular deposition of neurofibrillary tangles consisting of hyperphosphorylated microtubule-associated tau protein are considered to be critical mediators of AD pathophysiology [1,2,3]. AD is a multifactorial disease involving the central nervous system (CNS) [4,5]. Several hypotheses contributing to disease mechanisms have been proposed in addition to abnormal deposition of Aβ and tau protein such as mitochondria dysfunction, neurovascular compromise, cholinergic neuron damage, neuroinflammation, oxidative stress, glucose hypometabolism, and bacterial dysbiosis [6,7,8]. Evidence from animal models and human studies suggests that metabolic alterations such as impaired cerebral glucose metabolism [9] and brain insulin resistance [10,11] also play an important role in AD progression [12]. In fact, AD is suggested to have been associated with metabolic syndrome [13]. 

Cholesterol metabolism may also play an essential role in the pathophysiology of AD [14]. From human epidemiological studies, high levels of total cholesterol in serum could influence the processing of amyloid precursor protein (APP) in neurons, thereby increasing the risk for AD [15,16,17]. The activity of β- and γ-secretase complexes involved in APP cleavage depends on cholesterol metabolism [18]. Bile acids are amphipathic end-products of cholesterol metabolism, and dysregulation of their synthesis or metabolism is believed to play a role in hepatic, intestinal, metabolic, and various CNS diseases [19]. Bile acids are synthesized in the liver via two major pathways, the classical pathway, responsible for producing the primary bile acid cholic acid and chenodeoxycholic acid and the alternative pathway, responsible for producing the primary bile acid, chenodeoxycholic acid. Synthesis occurs through several enzymatic reactions catalyzed mainly by cytochrome P50s (CYPs). Animal models and clinical studies have reported anti-apoptotic, anti-inflammatory, and antioxidant effects of ursodeoxycholic acid and tauroursodeoxycholic acid in various neurologic diseases [20,21]. Recent studies also suggest primary and secondary bile acids influence AD pathophysiology [22]. We have also reported an altered brain and serum bile acid profile in the *App^NL-G-F^* mouse model of AD [23]. However, the specific liver cholesterol and bile acid metabolism changes in AD are unclear. 

This study was performed to better understand the role of lipid metabolism, particularly bile acid homeostasis, in the pathophysiology of AD. We evaluated the bile acid profile, including primary and secondary conjugated and unconjugated bile acids, in liver samples of male and female control and AD animals. Two well-characterized mouse models of AD, APP/PS1 and *App^NL-G-F^* mice were compared to examine the bile acid profile across sex or genotype. Our previous work demonstrated sex- and strain-specific changes in the brains and intestines of APP/PS1 and *App^NL-G-F^* mice [24]. Therefore, we elected to continue using both AD models to study liver metabolism and bile acid changes during disease.

## 2. Results

### 2.1. Primary and Secondary Liver Bile Acid Profile in Female APP/PS1 and App^NL-G-F^ Mice

**Figure 1:**
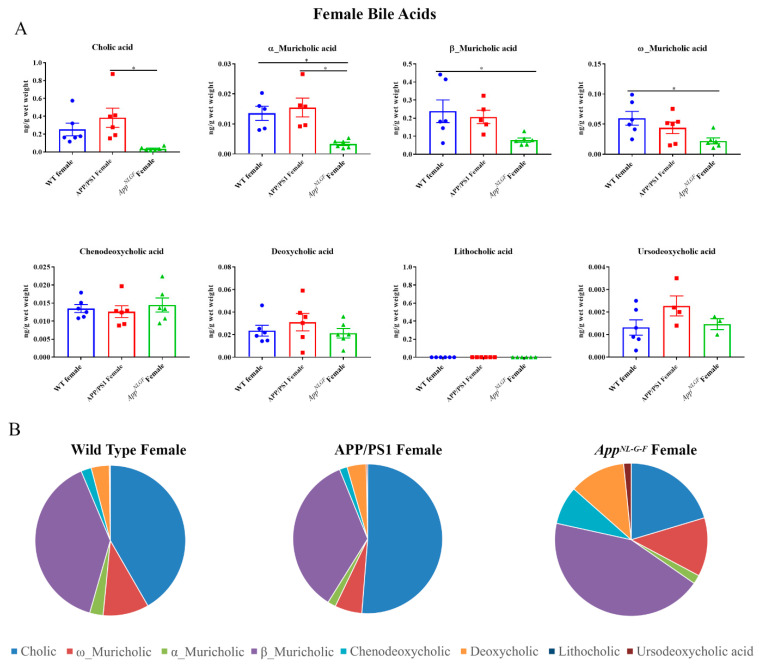
Primary and secondary liver bile acid levels in WT, APP/PS1, and *App^NL-G-F^* female mice. Livers were collected from wild-type (WT), APP/PS1, and *App^NL-G-F^* female mice at 6 months of age. Bile acids were quantified from liver lysates using ultra-performance liquid chromatography-tandem mass spectrometry (UPLC-MS/MS). (**A**) Data are presented as mean ± SEM. Significant differences were determined by one-way ANOVA, * *p* < 0.05 (*n* = 6) and outlier removal resulted in statistical analysis from (*n* = 5–6). (**B**) Relative comparisons of bile acid levels are also demonstrated as pie charts.

Both primary and secondary bile acids were quantified by ultra-performance liquid chromatography-tandem mass spectrometry (UPLC-MS/MS) from livers of female wild-type (WT), APP/PS1, and *App^NL-G-F^* mice. Primary bile acids α, β, and ω-muricholic acid were decreased in *App^NL-G-F^* mice compared to controls (Figure 1). On the other hand, no changes between groups were observed in concentrations of chenodeoxycholic acid and other secondary bile acids, including deoxycholic acid, lithocholic acid, and ursodeoxycholic acid, suggesting an alteration in primary bile acids in *App^NL-G-F^* and not APP/PS1 mice.

### 2.2. Conjugated Primary and Secondary Liver Bile Acid Profile in Female APP/PS1 and App^NL-G-F^ Mice

**Figure 2:**
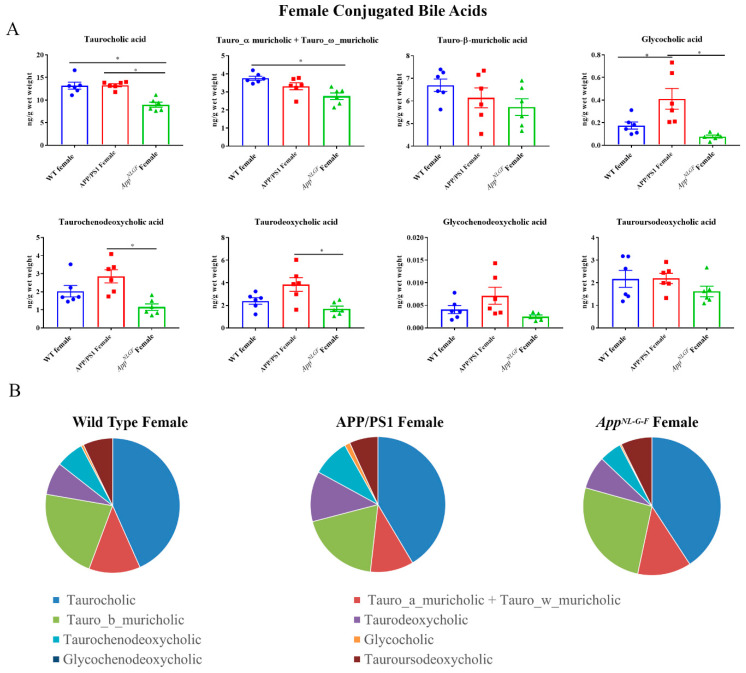
Conjugated bile acid levels in WT, APP/PS1, and *App^NL-G-F^* female mice. The levels of conjugated primary and secondary liver bile acids were quantified from 6-month-old wild-type (WT), APP/PS1, and *App^NL-G-F^* female mice using ultra-performance liquid chromatography-tandem mass spectrometry (UPLC-MS/MS). (**A**) Data are presented as mean ± SEM. Significant differences were determined by one-way ANOVA, * *p* < 0.05 (*n* = 6). (**B**) Relative comparisons of bile acid levels are also demonstrated as pie charts.

Conjugated primary and secondary bile acids were next quantified by ultra-performance liquid chromatography-tandem mass spectrometry from livers of female WT, APP/PS1, and *App^NL-G-F^* mice. Decreased concentrations of taurocholic acid and tauro-α, ω-muricholic acid were observed in *App^NL-G-F^* mice compared to WT controls (Figure 2). In contrast, glyco-conjugated cholic acid was increased in APP/PS1 mice compared to controls. These findings suggested a reduction in conjugated primary bile acid production occurred in *App^NL-G-F^* but not APP/PS1 mice.

### 2.3. Primary and Secondary Liver Bile Acid Profile in Male APP/PS1 and App^NL-G-F^ Mice

**Figure 3:**
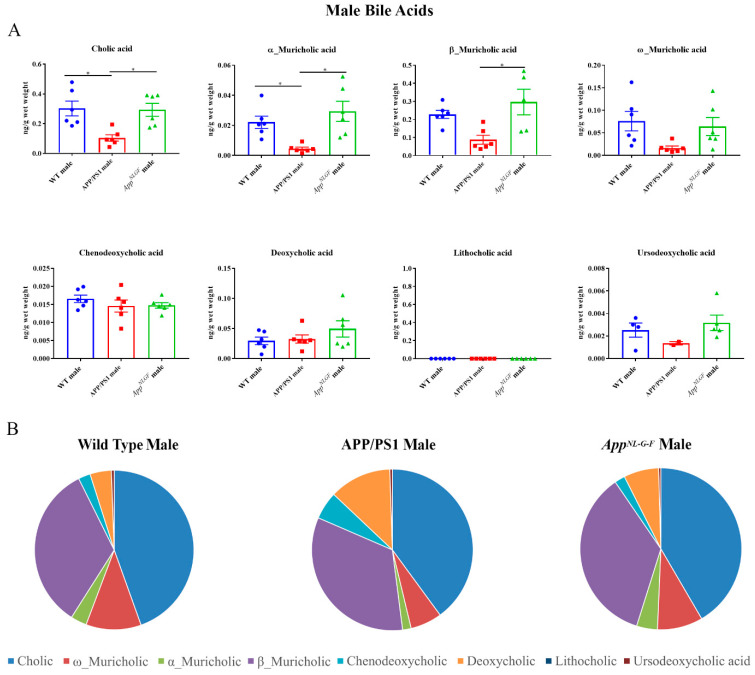
Primary and secondary liver bile acid levels in 6-month-old wild-type (WT), APP/PS1, and *App^NL-G-F^* male mice were quantified using ultra-performance liquid chromatography-tandem mass spectrometry (UPLC-MS/MS). (**A**) Data are presented as mean ± SEM. Significant differences were determined by one-way ANOVA, * *p* < 0.05 (*n* = 6). (**B**) Relative comparisons of bile acid levels are also demonstrated as pie charts.

To compare to females, primary and secondary bile acids were also quantified by ultra-performance liquid chromatography-tandem mass spectrometry from livers of male WT, APP/PS1, and *App^NL-G-F^* mice. Interestingly, APP/PS1 males had decreased levels of primary bile acids, cholic acid, and α-muricholic acid, compared to controls (Figure 3). No changes were observed in secondary bile acids in either of the AD mouse lines. 

### 2.4. Conjugated Primary and Secondary Liver Bile Acid Profile in Male APP/PS1 and App^NL-G-F^ Mice

**Figure 4:**
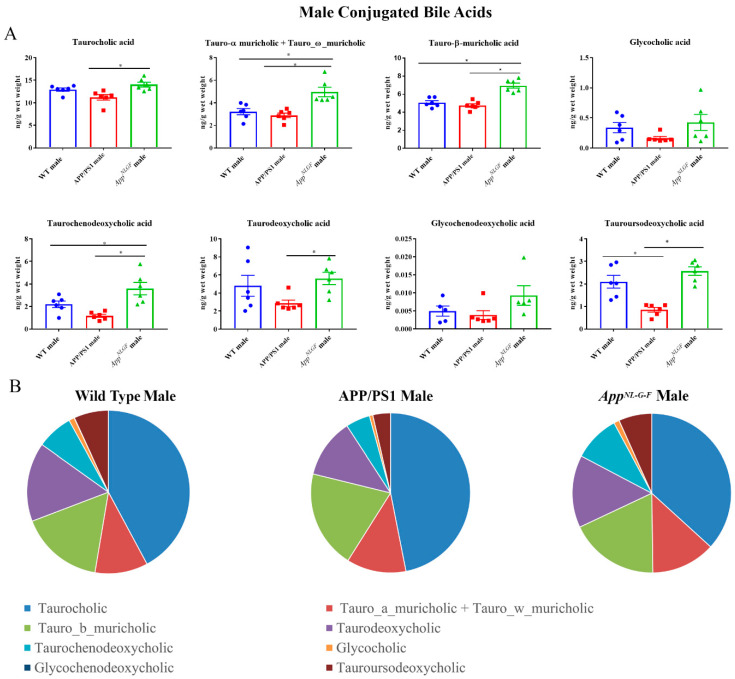
Conjugated bile acid levels in WT, APP/PS1, and *App^NL-G-F^* male mice. The levels of conjugated primary and secondary liver bile acids in 6-month-old wild-type (WT), APP/PS1, and *App^NL-G-F^* male mice were quantified using ultra-performance liquid chromatography-tandem mass spectrometry (UPLC-MS/MS). (**A**) Data are presented as mean ± SEM. Significant differences were determined by one-way ANOVA, * *p* < 0.05 (*n* = 6). (**B**) Relative comparisons of bile acid levels are also demonstrated as pie charts.

Conjugated primary and secondary bile acids were next quantified by ultra-performance liquid chromatography-tandem mass spectrometry from livers of male WT, APP/PS1, and *App^NL-G-F^* mice. Increases in concentrations of the conjugated primary bile acids tauro-α, ω-muricholic acid, tauro-β-muricholic acid, and taurochenodeoxycholic acid were observed in *App^NL-G-F^* mice compared to WT controls (Figure 4). In contrast, tauroursodeoxycholic acid, which has been shown to have neuroprotective properties, was decreased in APP/PS1 mice compared to their respective controls. 

### 2.5. Cholesterol and Bile Acid Intermediates in Livers of Male and Female APP/PS1 and App^NL-G-F^ Mice

**Figure 5:**
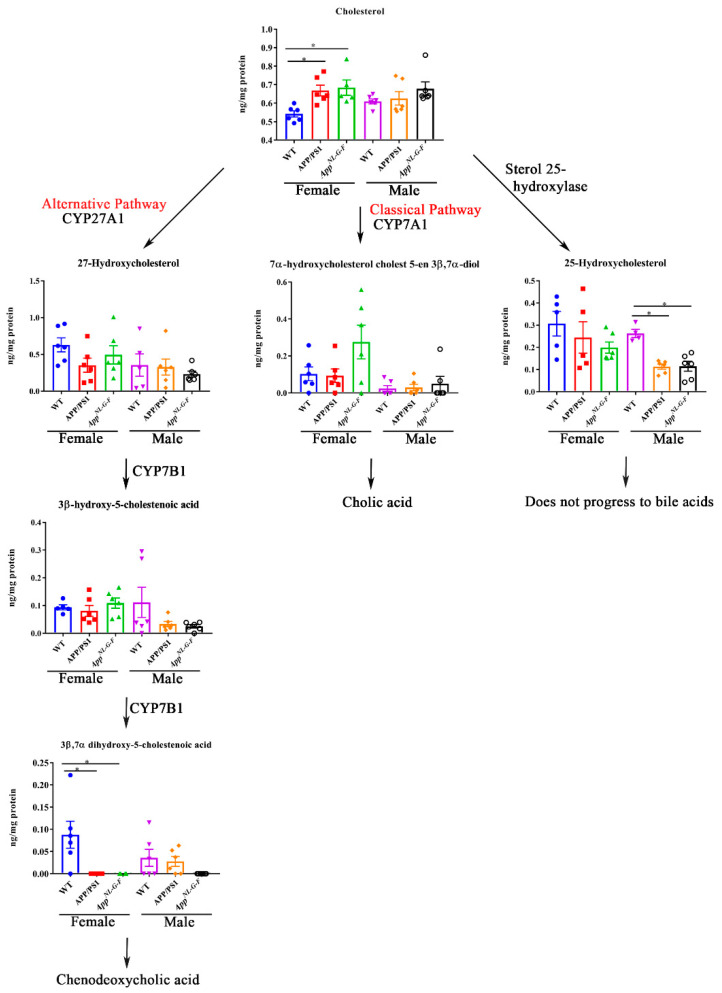
Bile acid synthesis intermediates in WT, APP/PS1, and *App^NL-G-F^* mice. Intermediates of bile acid synthesis in livers from 6-month-old wild-type (WT), APP/PS1, and *App^NL-G-F^* male and female mice were analyzed using ultra-performance liquid chromatography-tandem mass spectrometry (UPLC-MS/MS). Data are presented as mean ± SEM. Significant differences were determined by one-way ANOVA, * *p* < 0.05 (*n* = 6 and outlier removal resulted in statistical analysis from *n* = 5–6).

To investigate whether pathways of bile acid synthesis were altered in AD animals, the levels of cholesterol and bile acid intermediates including 27-hydroxycholesterol, 25-hydroxycholesterol, 7-α-hydroxycholesterol cholest5-en3β,7α-diol, 3β-hydroxy-5-cholestenoic acid, and 3β,7α-dihydroxy-5-cholestenoic acid were quantified by ultra-performance liquid chromatography-tandem mass spectrometry in WT, APP/PS1, and *App^NL-G-F^* female and male mice. Interestingly, cholesterol levels were increased in both APP/PS1 and *App^NL-G-F^* female mice, correlating with decreased 3β,7α-dihydroxy-5-cholestenoic acid levels (Figure 5). No differences in cholesterol levels were observed in male mice. Surprisingly, the levels of 25-hydroxycholesterol were reduced in both APP/PS1 and *App^NL-G-F^* male mice compared to controls (Figure 5).

### 2.6. Changes in Proteins Associated with Bile Acid Synthesis and Signaling in Female Mice

**Figure 6:**
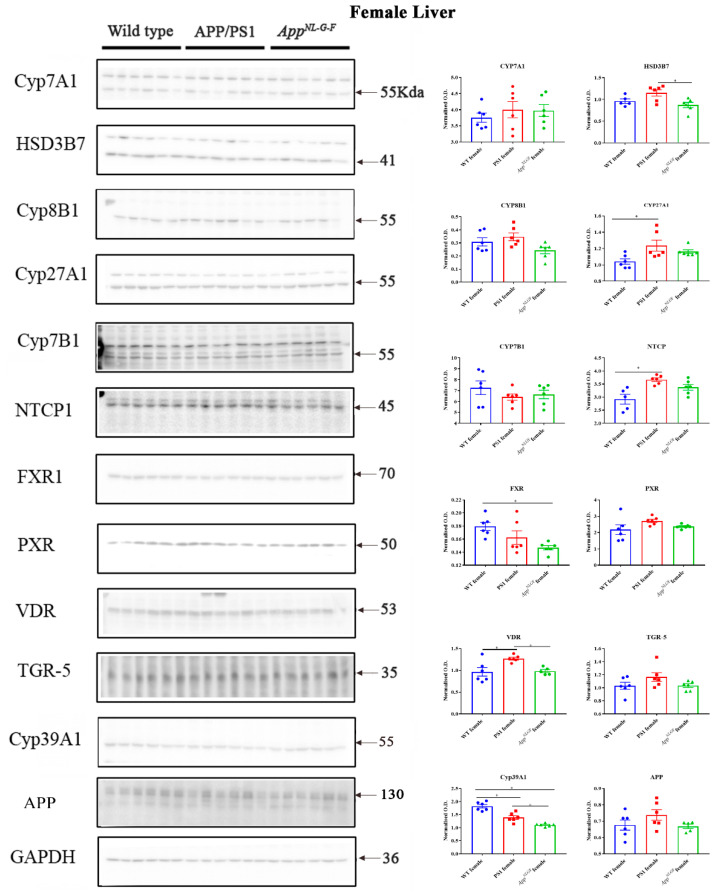
Bile acid synthesis and signaling proteins in WT, APP/PS1, and *App^NL-G-F^* female mice. Western blotting was performed from liver samples of 6-month-old wild-type (WT), APP/PS1, and *App^NL-G-F^* mice to evaluate protein levels of various enzymes involved in bile acid synthesis or signaling. Optical densities of proteins of interest were normalized against their respective GAPDH loading control. Data are presented as mean ± SEM. Significant differences were determined by one-way ANOVA, * *p* < 0.05 (*n* = 6 and outlier removal resulted in statistical analysis from *n* = 5–6).

To examine whether the altered bile acid composition and intermediates revealed in the female mice correlated with differences in levels of biosynthetic enzymes or bile acid signaling proteins, western blot analysis of liver lysates was performed. Protein levels of CYP7A1, HSD3B7, CYP8B1, CYP27A1, NTCP, FXR, PXR, VDR, TGR-5, CYP46A1, CYP39A1, APP, and CYP7B1 were compared in liver samples of WT, APP/PS1, and *App^NL-G-F^* female mice. We observed increased protein levels of NTCP, CYP27A1, and VDR and decreased CYP39A1 in APP/PS1 mice (Figure 6). In contrast, protein levels of FXR and CYP39A1 were decreased in *App^NL-G-F^* mice compared to controls (Figure 6). Levels of CYP27A1 were increased in the brains of only *App^NL-G-F^* mice (Supplementary Figure S1).

### 2.7. Changes in Proteins Associated with Bile Acid Synthesis and Signaling in Male Mice

**Figure 7:**
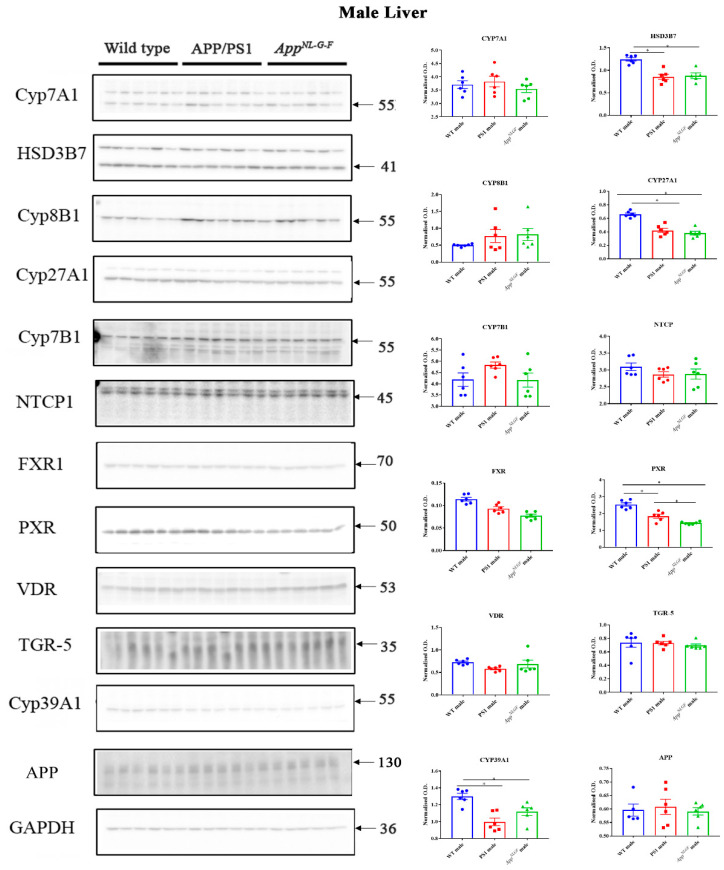
Bile acid synthesis and signaling proteins in WT, APP/PS1, and *App^NL-G-F^* male mice. Western blotting was performed from liver samples of male C57BL/6, APP/PS1, and *App^NL-G-F^* mice to evaluate protein levels of various enzymes involved in bile acid synthesis or signaling. Optical densities of proteins of interest were normalized against their respective GAPDH loading control. Data are presented as mean ± SEM. Significant differences were determined by one-way ANOVA, * *p* < 0.05 (*n* = 6 and outlier removal resulted in statistical analysis from *n* = 5–6).

A similar comparison of protein changes was performed on liver lysates from male mice. Protein levels of CYP7A1, HSD3B7, CYP8B1, CYP27A1, NTCP, FXR, PXR, VDR, TGR-5, CYP46A1, CYP39A1, APP, and CYP7B1 were measured in liver samples of male WT, APP/PS1, and *App^NL-G-F^* mice using western blotting. We observed a decrease in HSD3B7, CY27A1, PXR, and CYP39A1 in both APP/PS1 and *App^NL-G-F^* mice compared to controls (Figure 7). On the other hand, FXR and VDR levels were decreased only in *App^NL-G-F^* mice (Supplementary Figure S2).

## 3. Discussion

Our mass spectrometry profiling of bile acids and their intermediates revealed significant differences in the levels of conjugated and unconjugated liver primary bile acids in both APP/PS1 and *App^NL-G-F^* mice compared to controls. This change suggested that the overall liver metabolic profile is compromised as a function of disease. Additionally, we observed sex differences in liver cholesterol levels in both APP/PS1 and *App^NL-G-F^* mice, where increased cholesterol was found only in females and not males. This differential expression was most likely related to differences in levels of cytochrome P450 (CYP) enzymes. The decreased levels of 3beta,7alpha-dihydroxy-5-cholestenoic acid, an intermediate in “acidic” bile acid synthesis pathways, along with altered CYP enzymes, further support that altered liver metabolism or abnormal bile acids metabolism might be involved in the pathophysiology of AD. However, future studies are required to investigate the direct or indirect relationship of mutant APP expression or Aβ production with bile acid metabolism and any contribution to cognitive changes in AD.

One of the strategies to increase cholesterol excretion from the body is to decrease bile acid reabsorption in the intestine to promote a compensatory increase in bile acid synthesis from cholesterol that is excreted with feces [25]. Cholic, and chenodeoxycholic, and muricholic acids are the primary unconjugated bile acids derived from cholesterol by a sequence of enzymatic reactions occurring mainly in the liver and are later conjugated with taurine or glycine for secretion into bile. Despite an increase observed in female APP/PS1 and *App^NL-G-F^* mice cholesterol levels, we did not observe increased unconjugated bile acid synthesis in their livers. Instead, a decrease in primary bile acids was observed. This decrease could be due to the activity of other cholesterol elimination pathways, such as the formation of high-density lipoprotein (HDL), 22-hydroxycholesterol, pregnenolone, etc., in the AD mice, or their rapid conversion into conjugated forms followed by excretion with feces. Another reason for the decreased bile acids in the liver might be altered fecal bile acid excretion in these mice.

On the other hand, male *App^NL-G-F^* mice had increased conjugated primary bile acids, especially taurine-conjugated bile acids, including tauro-α,β,ω-muricholic acid, and taurochenodeoxycholic acid. After the synthesis of bile acids from cholesterol in the liver, they are conjugated with glycine or taurine before secretion into the bile and small intestine [26]. Elevated bile acids in the circulation usually result from increased bile acid synthesis and liver dysfunction [27]. Disturbances in bile acid metabolism can be caused by defective biosynthesis from cholesterol or imperfect conjugation. Here, an increase in taurine conjugation could be associated with an alteration in bile acid CoA synthase and bile acid CoA-amino acid N-acetyltransferase (BAAT), the enzyme involved in conjugation. A recent study using a cohort of subjects with AD and normal controls showed a marked increase in bile acids such as cholic acid and chenodeoxycholic acid compared to the healthy controls suggesting altered bile acid profiles might be contributing to AD pathophysiology [28]. There are numerous studies supporting critical biology for bile acids in the brain. For example, bile acid supplementation reportedly decreases inflammation and apoptosis during neuropathological states [19,29]. The interaction of bile acids with their receptors has been shown to regulated neurotransmission [30]. Chenodeoxycholic acid and other bile acids are natural ligands for nuclear receptor farnesoid X receptor (FXR) and deletion of FXR impairs memory and modulates neurotransmitter levels of GABA, glutamate, norepinephrine, and serotonin [31]. Bile acids are also known to induce signaling in ventral midbrain leading to enhance neurogenesis [32]. Moreover, studies from the Alzheimer’s Disease Neuroimaging Initiative cohort showed a strong association between bile acid profiles and Aβ, tau, and neurodegeneration biomarkers [22]. Another recent study using metabolic network analysis suggests that taurine transport, bile acid synthesis, and cholesterol metabolism differ in AD and cognitively normal individuals [33]. 

The conversion of cholesterol to bile acids involves hydroxylation, a saturation of the double bonds at C5–C6, epimerization of the 3-hydroxyl group, and oxidative cleavage of a 3-carbon unit from the side chain [34]. We assessed the levels of various enzymes involved and the intermediates formed during bile acid synthesis to better understand the mechanisms responsible for altered bile acid levels in APP/PS1 and *App^NL-G-F^* mice. Mass spectrometry analysis showed that 3beta,7alpha-dihydroxy-5-cholestenoic, the intermediate formed during the formation of chenodeoxycholic acid in the liver via the “acidic” pathway, was decreased in female APP/PS1 and *App^NL-G-F^* mice. However, no effects on the levels of chenodeoxycholic acid were observed, suggesting the involvement of the alternative pathways of BA synthesis in AD mice. In addition, we found that conjugated and unconjugated bile acids were altered not only in the AD females where the cholesterol levels were high but also in male APP/PS1 mice. This finding showed sex differences in bile acid synthesis in both AD models independent of liver cholesterol levels. Previous studies have shown sex differences in Aβ accumulation, insulin sensitivity, inflammation, plasma, and liver metabolic profile of APP/PS1 mice [35,36]. Studies have also shown differential amyloidosis and inflammatory responses in male and female *App^NL-G-F^* mice [37,38]. Estrogen signaling is known to regulate bile acid and cholesterol homeostasis. Hence, sex hormones should also be considered a critical component likely contributing to sex differences in bile acid composition [39]. A recent report showed a significant increase in hepatic and serum concentrations of bile acids after estrogen treatment and the elevated hepatic bile acid concentrations have been suggested to be caused by estrogen-related impairment in FXR activity [40]. Therefore, crosstalk between bile acid/FXR and estrogen/Erα signaling pathways may play a crucial role in the regulation of bile acid homeostasis and estrogen metabolism and hence could lead to sex differences in cholesterol levels and bile acid composition. 

Although bile acids are involved in lipid digestion and cholesterol homeostasis in the liver, their role in the mammalian system is much broader, and they are now recognized as critical signaling molecules with systemic endocrine functions [41]. Defective membrane transport in hepatocytes or alteration in the nuclear receptors such as PXR, FXR, and VDR can affect bile acid secretion or metabolism and control liver inflammation [42]. We observed increased CYP27A1, NTCP, and VDR and decreased CYP39A1 in APP/PS1 females, while reduced FXR and CYP39A1 were found in *App^NL-G-F^* female mice. Physiologic concentrations of chenodeoxycholic acid and cholic acid are endogenous agonists for FXR. On the other hand, activation of FXR by bile acids induces a negative feedback mechanism by inhibiting transcription of bile acid genes in hepatocytes or inhibits the enzymes that synthesize bile acids from cholesterol precursors [43,44]. A reduction in FXR in *App^NL-G-F^* female mice might be associated with a decrease in both conjugated and unconjugated muricholic acid in these animals. VDR regulates calcium homeostasis, immunity, cellular differentiation and has also been known to reduce bile acid synthesis and therefore is a negative-feedback regulator of bile acid synthesis [45]. VDR is associated with the hepatic activity of CYP27A1 and often also with hepatic injury [46]. We observed an increase in VDR and CYP27A1 in female APP/PS1 mice compared to controls suggesting an impairment in female AD mice liver function. In contrast, both APP/PS1 and *App^NL-G-F^* male mice showed reduced HSD3B7, CYP27A1, CYP39A1, and PXR levels. PXR is also known to regulate bile acid detoxification and has been suggested as a therapeutic agent for treating chronic cholestatic liver disease by regulating bile formation and secretion [47,48]. Decreased PXR in APP/PS1 and *App^NL-G-F^* male mice might result from liver inflammation in these AD animals. 

It is interesting to note that no significant changes were observed in secondary bile acids in male or female APP/PS1 and *App^NL-G-F^* mice compared to controls. Secondary bile acids, deoxycholic acid, and lithocholic acid are formed through bacterial 7α-dehydroxylation of the primary bile acids, cholic acid, and chenodeoxycholic acid, respectively, in the colon [49]. Thus, the composition of secondary bile acids can be profoundly affected by microbial community structure and function and gut dysbiosis can alter secondary bile acids [50]. Lithocholic acid was below detection limits in all samples and no significant differences were observed in deoxycholic levels. Only taurourosdeoxycholic acid was decreased in male APP/PS1 mice when compared to controls. This bile acid is implicated in a range of beneficial metabolic effects such as reducing insulin resistance and diabetes, being a neurological protection agent, a treatment of cholelithiasis cholestatic liver disease, and attenuating amyloid precursor protein processing and Aβ deposition in a mouse model of AD [51,52,53]. A reduction of this bile acid selectively in APP/PS1 but not *App^NL-G-F^* male mice suggests a genotype-associated bile acid alteration.

It is well known that most of the bile acids are reabsorbed in the ileum and are transported back to the liver via portal blood circulation/enterohepatic circulation to inhibit bile acid synthesis mainly via CYP7A1 activity, which is regulated by several members of the nuclear receptor superfamily of ligand-activated transcription factors in the liver [34,54]. In our study, no significant changes were observed in the protein expression of enzymes involved in bile acid formation including the rate-limiting enzyme CYP7A1 which could be due to the non-availability of other receptors such as 9-cis retinoic acid receptor-alpha (RXRα;NR2B1) required for the formation of a regulatory cascade of nuclear receptors to regulate bile acid synthesis. The other reason for a decrease of bile acid in the liver could be less availability of cholesterol as a substrate or precursor for bile acid synthesis. Cholesterol can be directly converted to steroid hormones or other derivatives apart from their conversion to bile acids. The intestine serves as the site of both the absorption of dietary cholesterol and reabsorption of biliary cholesterol or excretion of excess cholesterol which regulates the balance of cholesterol [55]. It has also been suggested that the half-life of bile acid is largely dependent on factors in the gut rather than the liver [56]. Unfortunately, we had not collected intestines from these animals to analyze bile acids composition, which is one of the drawbacks of our study.

In conclusion, these findings demonstrate that there exist sex-dependent differences in cholesterol metabolism in the liver. Our data also indicates differential bile acid metabolism in male and female APP/PS1 and *App^NL-G-F^* mice. This is the first study reporting a difference in liver bile acids in AD mouse models to the best of our knowledge. This work supports the idea that changes in liver bile acid metabolism may be a component of a systemic manifestation of AD. However, future work will be needed to determine whether the liver changes express any temporal profile and correlate with any brain or behavior changes.

## 4. Materials and Methods

### 4.1. Animals 

*App^NL-G-F^* mice were obtained from Dr. Takaomi C. Saido, Laboratory for Proteolytic Neuroscience, RIKEN Center for Brain Science, Japan. APP is not overexpressed in *App^NL-G-F^* mice. Pathogenic Aβ is elevated due to effects from 3 mutations associated with familial AD. Specifically, an APP construct containing a humanized Aβ region including the Swedish “NL,” the Iberian “F,” and the Arctic “G” mutations was used [57]. This model was selected to avoid potential confounds introduced by APP over-expression or integration artifacts. The APP/PS1 transgenic mice (APPSwe/PSEN1dE9) and littermate control wild-type (WT) mice (C57BL/6J) were obtained from the Jackson Laboratory. APP/PS1 express the Swedish mutation in APP and dE9 mutation in the PS1 gene, resulting in human APP expression and secretion of human Aβ [58]. Both males and females from all three strains of mice, C57BL/6J (WT), APP/PS1, and *App^NL-G-F^*, were used. 

### 4.2. Animal Use

The animals were provided food and water *ad libitum* and housed with a 12-h light/dark cycle. At 6 months of age, mice were euthanized by CO_2_ asphyxiation followed by cardiac perfusion with PBS. Brains and livers were collected and flash-frozen in liquid nitrogen for biochemical analyses.

### 4.3. Antibodies and Reagents

Antibodies against CYP7A1 (cat no: ab65596, 1:400), HSD3B7 (cat. no: ab190223, 1:1000), CYP8B1 (cat.no: ab191910, 1:1000), CYP27A1 (cat.no: ab126785, 1:2000), NTCP (cat.no: ab131084), farnesoid X receptor (FXR) (cat.no: ab155124, 1:1000), pregnane X receptor (PXR) (cat.no: ab192579, 1:1000), vitamin D receptor (VDR) (cat.no:, ab3508, 1:700), Takeda G-protein coupled receptor 5 (TGR-5) (cat. no: ab72608, 1:1000), Anti-amyloid precursor protein (Y188) (cat. no: ab32136, 1:750) and CYP46A1 (cat.no: ab244241, 1:1000) were obtained from Abcam (Cambridge, MA, USA). Anti-CYP39A1 antibody (cat.no MBS3223750, 1:500) was purchased from MyBioSource (MyBioSource, Inc, San Diego, CA, USA), and anti-CYP7B1 antibody (cat no: H00009420-A01, 1:750) was obtained from Novus Biologicals (Novus Biologicals, LLC, Centennial, CO, USA). Anti-glyceraldehyde 3-phosphate dehydrogenase (GAPDH) antibody (cat. no: Sc-2064, 1:2000) and horseradish peroxidase-conjugated secondary antibodies were purchased from Santa Cruz Biotechnology (Santa Cruz, CA, USA). Standards used in mass spectroscopy analysis were purchased from MilliporeSigma. Autosampler vials were obtained from Thermo Fisher Scientific (Waltham, MA, USA). Silanized micro vial inserts were from Agilent (Santa Clara, CA, USA; part #5181e8872) and inserts were from VWR (Radnor, PA, USA).

### 4.4. Western Blot Analysis

Frozen liver samples were pulverized under liquid nitrogen temperatures. Temporal cortices and pulverized liver samples were used for western blotting. Lysates were prepared in RIPA buffer containing protease inhibitors using a Bullet Blender Storm homogenizer 24 (Next Advance, Inc, Troy, NY, USA) at medium speed followed by centrifugation (21,000× *g*, 4C, 10 min) to remove insoluble content. The protein content of the supernatants was determined using a BCA protein determination assay (Pierce Biotechnology, Rockford, IL, USA). The resultant supernatants were boiled in SDS containing gel-loading sample buffer for 5 min. Fifteen µg of each total protein extract was resolved on a 10% SDS polyacrylamide gel. Separated proteins were transferred onto polyvinylidene difluoride membranes for western blotting using anti-CYP7A1, HSD3B7, CYP8B1, CYP27A1, NTCP, FXR, PXR, VDR, TGR-5, CYP46A1, CYP39A1, CYP7B1, and GAPDH (loading control) antibodies. Bands were visualized using enhanced chemiluminescence (GE Healthcare, Piscataway, NJ, USA). Chemiluminescent images were captured using an Aplegen Omega Lum G Imaging System. Optical densities were quantified using Adobe Photoshop 12.0 software. Optical density values were normalized to their relevant loading control GAPDH optical density values from the same membrane.

### 4.5. Cholesterol Metabolites Analysis Using UPLC-MS/MS

UPLC-MS/MS analysis was performed using a Waters Acquity UPLC system coupled to a Waters Xevo TQ-S triple quadrupole mass spectrometer. Bile acids were analyzed as we previously described [23]. Briefly, liver samples were pulverized under liquid nitrogen temperatures, and ~10 mg of homogeneous powder were sonicated with 50 µL of 100% methanol containing a mixture of bile acid internal standards (1 ng of taurocholic-d_5_, cholic-d_4_, glycocholic-d_4_, and 10 ng of chenodeoxycholic-d_4_, Medical Isotopes, Pelham, NH, USA) as previously described [23]. After centrifugation at 12,000× *g* for 10 min, 10 µL of supernatants were used for UPLC-MS/MS analysis, while pellets were used for protein quantification. Bile acids were chromatographically resolved on a Waters ACQUITY UPLC HSS T3 column (1.8 μM, 100 Å pore diameter, 2.1 × 150 mm; Waters) with an ACQUITY UPLC HSS T3 precolumn (1.8 μM, 100 Å pore diameter, 2.1 × 5 mm; Waters) using a gradient of water with 0.1% formic acid and acetonitrile with 0.1% formic acid as we previously described [23]. Bile acids were monitored in the multiple reaction monitoring modes and quantified against deuterated internal standards as we previously described [23].

Liver cholesterol was extracted using methanol as we described for bile acids. One µg of cholesterol-d7 was added to each sample as an internal standard during extraction. Ten µL of supernatant was loaded onto a Waters ACQUITY UPLC HSS T3 column (1.8 μM, 100 Å pore diameter, 2.1 × 150 mm; Waters) with an ACQUITY UPLC HSS T3 precolumn (1.8 μM, 100 Å pore diameter, 2.1 × 5 mm; Waters). The elution program was a modification of a previously described method [59]. The flow rate was maintained at 0.2 mL/min. Initial solvent A (acetonitrile: water (40:60) with 10 µM ammonium acetate and 0.025% acetic acid) was 60%, and solvent B (acetonitrile: 2-propanol (10:90) containing 10 µM ammonium acetate and 0.02% acetic acid) was 40%. At 0.5 min, %B was increased to 99% over 10 min, and after 8 min was returned to 40% over 0.5 min. The column was equilibrated for 5 min between injections. Cholesterol (*m*/*z* = 369.4) and its internal standard d7-cholesterol (*m*/*z* = 376.8) were ionized with a positive electrospray ionization (ESI) technique and monitored in the selected ion monitoring mode as [M-OH]^+^.

Liver hydroxycholesterols were extracted using a two-phase acetone liquid/liquid extraction method [60,61] with the modification that the hexane fraction was collected for MS analysis. Briefly, ~10 mg of liver powder was homogenized in 3 mL of 2:1 acetone:saline with 1 ng of d5-27-hyrdoxycholesterol as an internal standard. The homogenate was centrifuged at 2000× *g* for 10 min, and the supernatant was extracted 3 times with 2 mL of hexane. The hexane extracts were dried down under nitrogen, re-dissolved in 30 µL of 50% acetonitrile, and 10 µL were analyzed on UPLC-MS/MS system. UPLC separation was achieved as we described for bile acids. MS/MS analysis was performed in a positive ESI using multiple reaction monitoring modes. The following mass transitions (with collision energies indicated in parentheses (V)) were used: 27-hyrdoxycholesterol 385.37/161.11 (24); 25-hyrdoxycholesterol 385.37/159.09 (24); 7α-hydroxycholesterol cholest 5-en 3β,7α-diol 385.37/367.35 (14); 3β-hydroxy-5-cholestenoic acid 399.35/161.11 (28); 3β,7α dihydroxy-5-cholestenoic acid 415.35/105.12 (44); d5-27-hyrdoxycholesterol 390.45/161.11 (24).

### 4.6. Statistical Analyses

Statistical analyses were performed using Graphpad Prism (Prism version 7.00 for Windows, GraphPad Software, La Jolla, CA, USA). The tests for outliers (Grubb’s test) and normality (Shapiro-Wilk test) were performed on each data set. If the data followed a normal distribution, a one-way ANOVA was performed to compare the three conditions. We have not compared males and females due to the small sample sizes and further studies will be needed to better compare sex differences in the different AD models. All plots in the manuscript were generated using GraphPad software. Results are presented as mean values ± the standard error of the mean (SEM). Differences were considered significant when *p* < 0.05 and indicated in the figure legend as appropriate.

### 4.7. Ethics Statement

The *App^NL-G-F^* mice were obtained from Dr. Takaomi C. Saido, Laboratory for Proteolytic Neuroscience, RIKEN Center for Brain Science, Japan. The APP/PS1 transgenic mice and littermate control wild-type (WT) mice (C57BL/6J) were obtained from the Jackson Laboratory. Animal use was approved by the University of North Dakota Institutional Animal Care and Use Committee (IACUC protocol # 1711-1C).

## Data Availability

All data generated or analyzed during this study are included in this published article and its supplementary information files.

## Supplementary Materials

The following are available online at https://www.mdpi.com/article/10.3390/ijms22147451/s1.

## Abbreviations

AD	Alzheimer’s disease
CYPs	Cytochrome P50s)
PS1	Presenilin
CYP7A1	Cytochrome P450 Family 1 Subfamily A Member 1
HSD3B7	Hydroxy-Delta-5-Steroid Dehydrogenase, 3 Beta- And Steroid Delta-Isomerase 7
CYP8B1	Cytochrome P450 Family 8 Subfamily B Member 1
CYP27A1	Cytochrome P450 Family 27 Subfamily A Member 1
NTCP	Sodium/taurocholate cotransporting polypeptide
FXR	Farsenoid X receptor
PXR	Pregnane X receptor
VDR	Vitamin D receptor
TGR-5	Takeda G-protein coupled receptor 5
CYP46A1	Cytochrome P450 Family 46 Subfamily A Member 1
APP	Amyloid precursor protein
UPLC-MS/MS	Ultra performance liquid chromatography—tandem mass spectrometer
MS	Mass spectrometer
BAAT	Bile acid CoA-amino acid N-acetyltransferase
