# Supplementary material for: Liver Bile Acid Changes in Mouse Models of Alzheimer’s Disease
Source: Int J Mol Sci. 2021 Jul 12;22(14):7451. doi: 10.3390/ijms22147451 (PMC8303891; doi:10.3390/ijms22147451)
Supplement: Supplementary file 1 [file ijms-22-07451-s001.zip › Sup figures IJMS/ijms-1215723-supplementary.pdf]

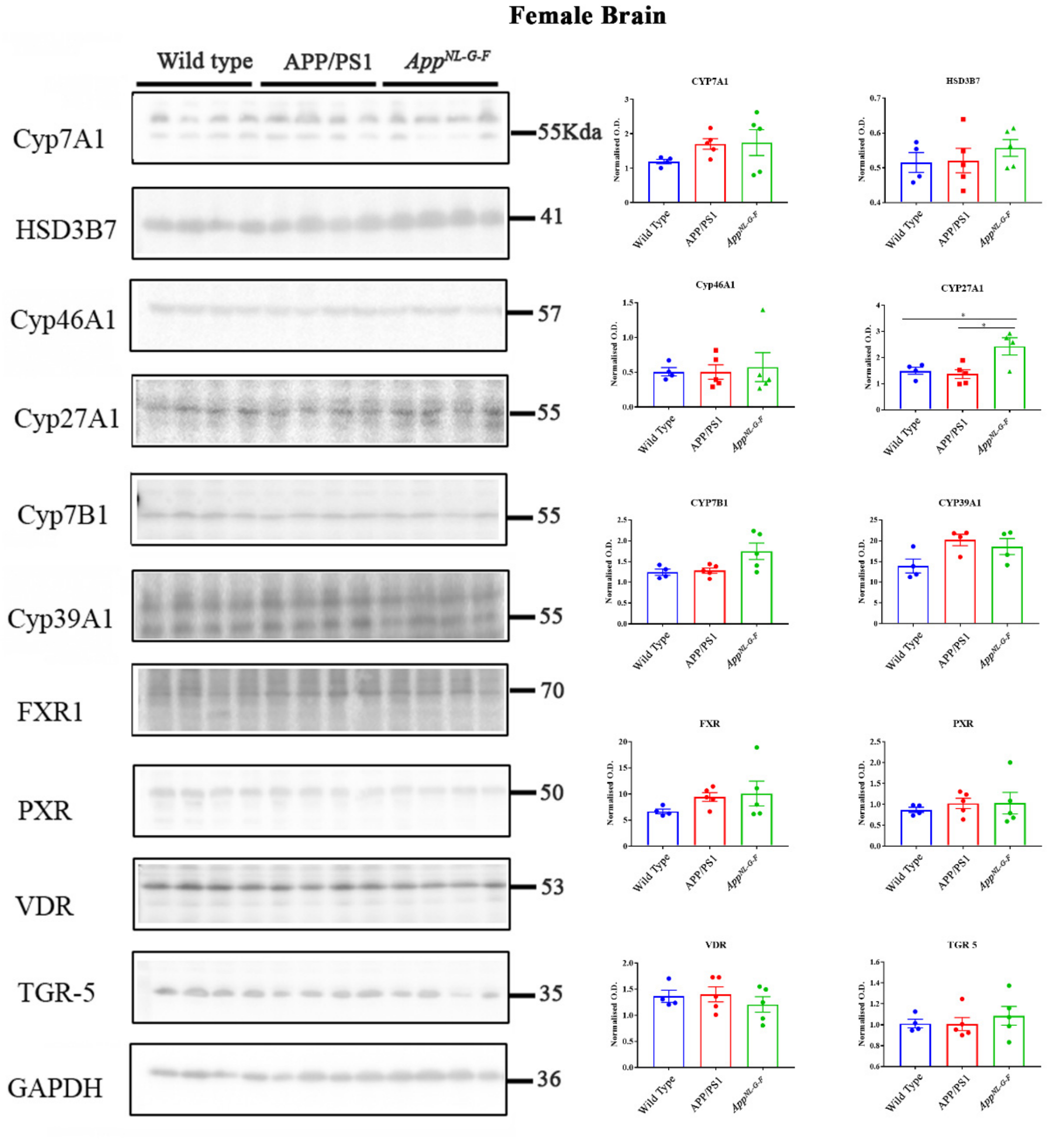

Supplementary Figure S1. Bile acid synthesis and signaling proteins in brains of WT, APP/PS1, and *App*$^{NL\text{-}G\text{-}F}$ female mice. Western blotting was performed from brain samples of female C57BL/6, APP/PS1, and *App*$^{NL\text{-}G\text{-}F}$ mice to evaluate the protein levels of various enzymes involved in bile acid synthesis or signaling. Optical densities of proteins of interest were normalized against their respective GAPDH loading control. Data are presented as mean ± SEM. Significant differences were determined by one-way ANOVA, * $p<0.05$ (n=6 and outlier removal resulted in statistical analysis from n=5-6).

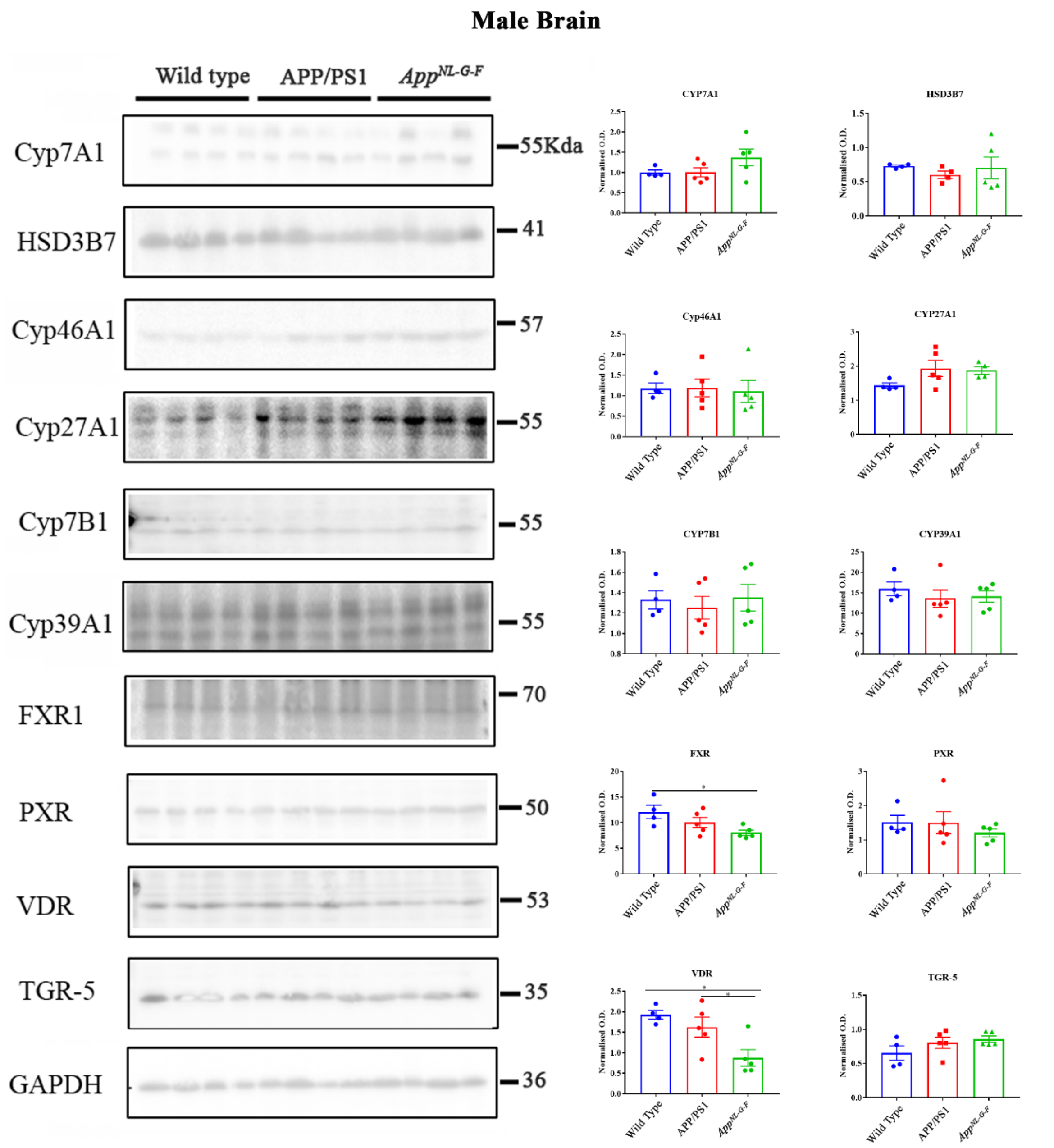

Supplementary Figure S2. Bile acid synthesis and signaling proteins in brains of WT, APP/PS1, and $App^{NL\text{-}G\text{-}F}$ male mice. Western blotting was performed from brain samples of male C57BL/6, APP/PS1, and $App^{NL\text{-}G\text{-}F}$ mice to evaluate the protein levels of various enzymes involved in bile acid synthesis or signaling. Optical densities of proteins of interest were normalized against their respective GAPDH loading control. Data are presented as mean ± SEM. Significant differences were determined by one-way ANOVA, * $p<0.05$ (n=6 and outlier removal resulted in statistical analysis from n=5-6).
