# Supplementary figures and images for: Liver Bile Acid Changes in Mouse Models of Alzheimer’s Disease
Source: Int J Mol Sci. 2021 Jul 12;22(14):7451. doi: 10.3390/ijms22147451 (PMC8303891; doi:10.3390/ijms22147451)

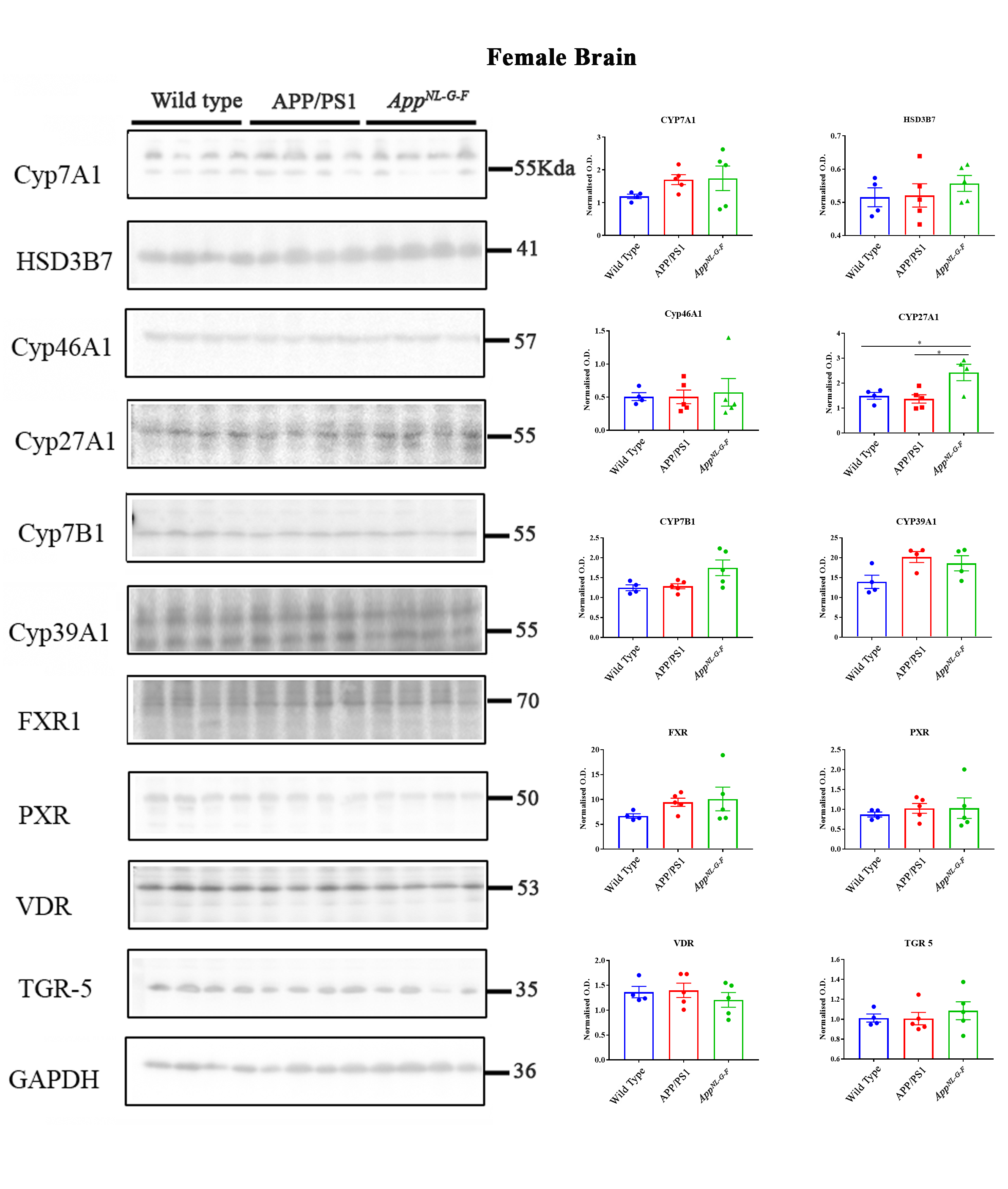

Supplement: Supplementary file 1 [file ijms-22-07451-s001.zip › Sup figures IJMS/Sup Figure S1 female brain western .tif]

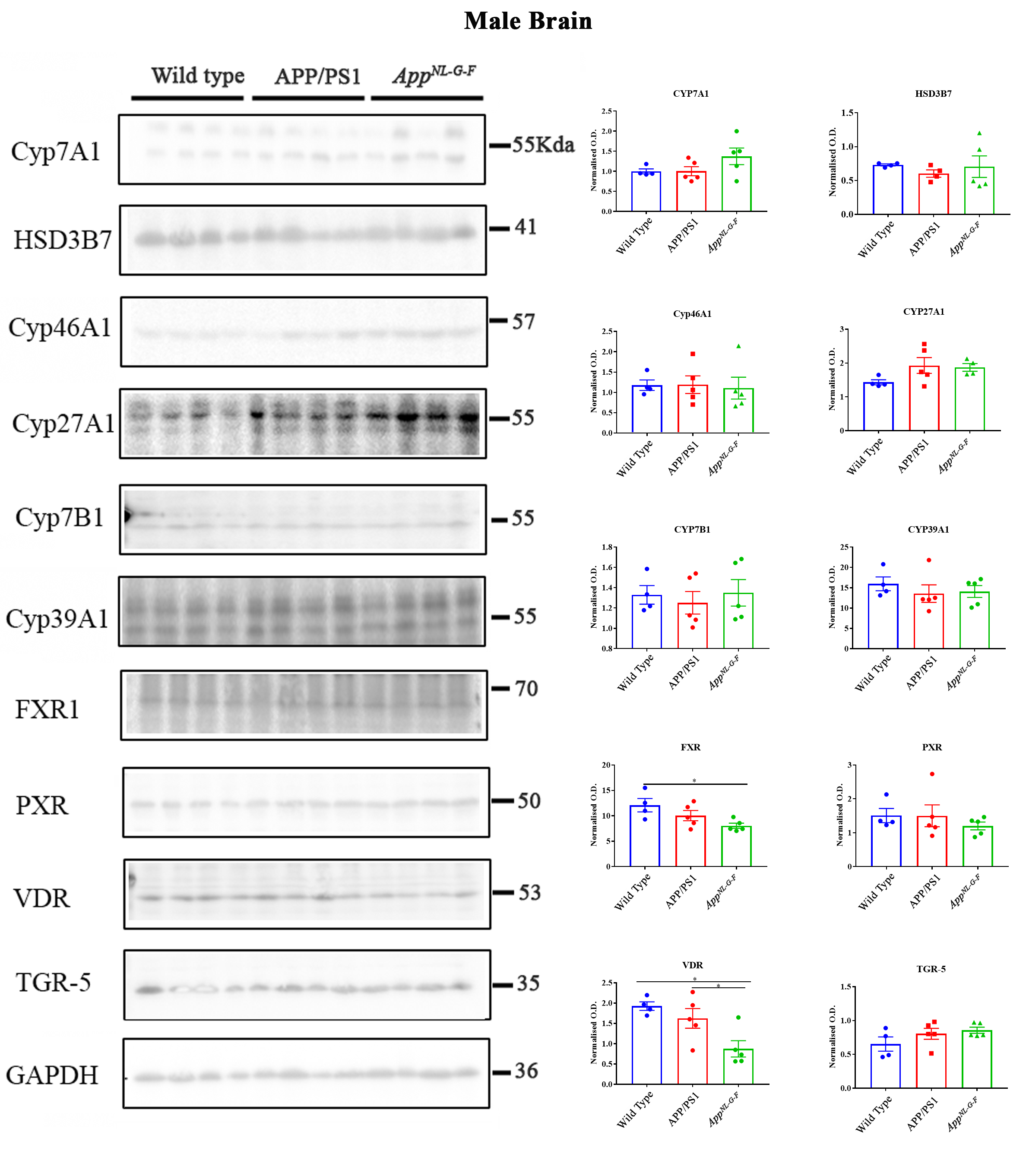

Supplement: Supplementary file 1 [file ijms-22-07451-s001.zip › Sup figures IJMS/Sup Figure S2 male brain western .tif]
